# PD44 Handbook Of Evidence-Based Practice In Psychology (EBPP): Addressing The Urgent Need For Educational Resources On EBPP In Portuguese

**DOI:** 10.1017/S0266462325101773

**Published:** 2025-12-29

**Authors:** Tamara Melnik

## Abstract

**Introduction:**

The primary objective of the Handbook of EBPP is to empower professionals, researchers, students, and the public with the skills needed to read and critically interpret biomedical literature relevant to psychology. By fostering a deeper understanding of evidence-based approaches, the Handbook aims to enhance the quality and effectiveness of mental health services, ultimately improving care outcomes.

**Methods:**

The Handbook is systematically organized into three main sections.

- Foundations: This section explores the conceptual and historical development of EBPP, establishing its relevance and importance within psychological practice.
- Methodology: Detailed guidance is provided on essential methodological tools, such as framing research questions with the PICO (population, intervention, comparator, outcome) model, navigating electronic databases effectively, and critically analyzing scientific literature. Emphasis is placed on the role of experimental designs, systematic reviews, and meta-analyses in evidence-based practice.
- Interventions: The third section focuses on the efficacy, effectiveness, and safety of psychosocial interventions, highlighting their critical role in the prevention and treatment of mental health disorders.

**Results:**

Although quantitative assessments of the Handbook’s impact are not included in this abstract, its comprehensive approach addresses a crucial gap in the Portuguese-speaking mental health community. By presenting accessible, scientifically validated practices, it establishes itself as a cornerstone for evidence-based psychological education and care.

**Conclusions:**

The Handbook of EBPP meets an urgent demand for Portuguese-language educational materials on evidence-based psychology. Adhering to the principles of the Cochrane Collaboration, it ensures that clinical guidelines are transparent, rigorously evaluated, and regularly updated. This resource is a significant step forward for the professionalization of psychological practice in Brazil, advancing ethical, evidence-based care and improving individual mental health outcomes.

